# A Novel Friendly Jamming Scheme in Industrial Crowdsensing Networks against Eavesdropping Attack

**DOI:** 10.3390/s18061938

**Published:** 2018-06-14

**Authors:** Xuran Li, Qiu Wang, Hong-Ning Dai, Hao Wang

**Affiliations:** 1 Faculty of Information Technology, Macau University of Science and Technology, Macau SAR, China; lxrget@163.com (X.L.); qiu_wang@foxmail.com (Q.W.); 2 Department of ICT and Natural Sciences, Faculty of Information Technology and Electrical Engineering, Norwegian University of Science and Technology, Postboks 1517, NO-6025 Aalesund, Norway

**Keywords:** friendly jamming, crowdsensing, industrial internet of things, security

## Abstract

Eavesdropping attack is one of the most serious threats in industrial crowdsensing networks. In this paper, we propose a novel anti-eavesdropping scheme by introducing friendly jammers to an industrial crowdsensing network. In particular, we establish a theoretical framework considering both the probability of eavesdropping attacks and the probability of successful transmission to evaluate the effectiveness of our scheme. Our framework takes into account various channel conditions such as path loss, Rayleigh fading, and the antenna type of friendly jammers. Our results show that using jammers in industrial crowdsensing networks can effectively reduce the eavesdropping risk while having no significant influence on legitimate communications.

## 1. Introduction

Crowdsensing is a technique leveraging the crowd power to accomplish sensing tasks collaboratively at a low cost. The participants in crowdsensing networks sense the information and upload the sensed data to crowdsensing platforms voluntarily. As a result, the quality of sensing tasks heavily relies on whether the number of participants is sufficient. However, due to the consumption on time, battery and data, recruiting the participants in crowdsensing networks is difficult, although some incentive mechanisms were proposed [1]. Therefore, to guarantee the sensing quality of accomplished tasks, mobile crowdsensing as the complement of traditional statically deployments has been extensively investigated [2,3].

In recent years, the combination of crowdsensing and Industrial Internet of Things (IIoT) has drawn extensive attention [4,5]. There are a lot of benefits in introducing crowdsensing to IIoT, including: (1) providing mobile and scalable measures; (2) monitoring new areas without installing additional dedicated devices; (3) integrating human wisdom into machine intelligence straight forwardly; (4) sharing information and making decision among the whole industrial community [4]. The performance of personal monitoring, process monitoring and product quality checking in IIoT will be improved with the help of crowdsensing [5].

With the proliferation of wireless sensor devices, the security of transmitting data in such IIoT based crowdsensing networks deserves much attention, especially for the confidential data related with commercial interest and privacy concern. To protect the security of crowdsensing networks, some security encryption schemes were proposed, such as privacy-preserving participant selection scheme [6] and reputation management schemes [7]. Moreover, some encryption schemes for IIoT were presented in [8]. The encryption schemes are feasible for devices with sufficient computing capability and power, such as smart phones or tablet computers. However, the encryption schemes may not be suitable for power-constraint sensor devices in crowdsensing networks (e.g., pulse-sensor-embedded wrists) and machinery in factories, since these schemes often require conducting compute-intensive tasks, consequently consuming a lot of power.

Different from the security encryption schemes, friendly-jamming schemes have been recognized as a promising approach to enhance the network security without bringing extra computing tasks [9,10,11,12,13]. The main idea of friendly-jamming schemes is introducing some friendly jammers to wireless networks, where these friendly jammers can generate a jamming signal to increase the noise level at the eavesdroppers, so that they cannot successfully wiretap the legitimate communications [14,15,16]. Using friendly-jamming schemes to decrease the possibility of eavesdropping attacks has received extensive attention [17,18]. The benefits of friendly jamming schemes is that there is no requirement for strong-computing capability of nodes, and no necessity for centralizing security schemes [19]. Therefore, friendly-jamming schemes can be applied in crowdsensing networks with power-constraint devices.

To mitigate the eavesdropping attack of crowdsensing networks, we propose a novel friendly jamming scheme in this paper. In this scheme, we place multiple jammers at a circular boundary around the protected communication area. Being implied by previous studies [20,21], we also consider equipping directional antennas at jammers. We name such jamming scheme with directional antennas as DFJ. Moreover, we also consider equipping omnidirectional antennas at jammers. We name such a jamming scheme with omnidirectional antennas as OFJ. For comparison purposes, we also consider the case without jammers (named as NFJ).

The main contributions of this paper are summarized as follows.

- We propose friendly jamming schemes (DFJ and OFJ) to protect confidential communications from eavesdropping attacks.
- We establish a theoretical model to analyze the *probability of eavesdropping attacks* and the *probability of successful transmission* to evaluate the effectiveness of our proposed scheme.
- We conduct extensive simulations to verify the accuracy of our theoretic model. The results also show that using jammers in crowdsensing networks can effectively reduce the eavesdropping risk while having no significant influence on legitimate communications.

Our proposed schemes have many more merits than other existing anti-eavesdropping schemes. Firstly, our schemes are less resource-intensive (i.e., no extensive computing resource needed) and it does not require any modifications on existing network infrastructure. Secondly, our schemes are quite general since the circular area with jammers can essentially circumscribe any buildings due to the feature that every simple polygon (i.e., the shape of a building) always has a circumscribed circle [22]. As a result, the effective jamming to eavesdroppers can be achieved. Moreover, our schemes can offer a larger effective protection area compared with other friendly jamming schemes like placing jammers at polygons [14] or other shapes [23] due to the largest coverage area of a circle.

## 2. System Models

In this section, we introduce the models used in this paper. We mainly focus on the uplink transmission from sensor devices (legitimate transmitter) to the receiver. The descriptions of notations are given in Table 1.

### 2.1. Network Model

**Figure 1:**
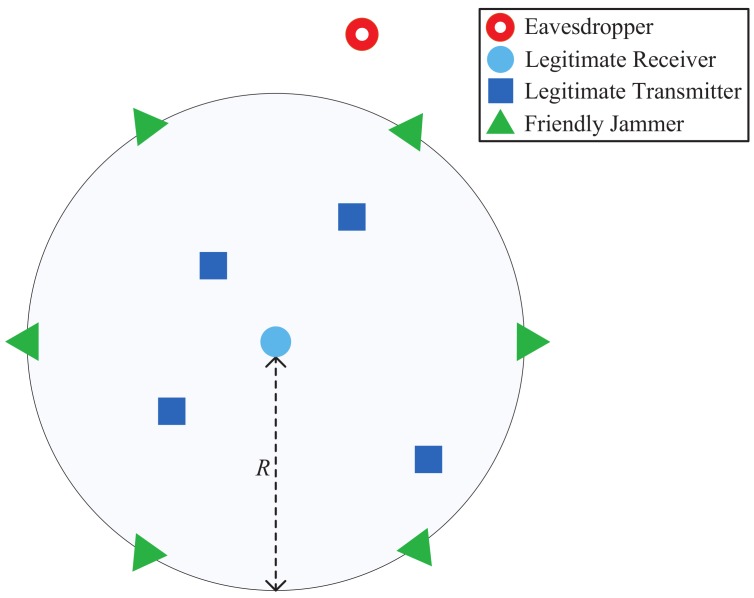
Network model.

In this paper, we consider a finite disk communication area with radius *R*, as shown in Figure 1. In this area, a number of legitimate transmitters are distributed according to Poisson point process (PPP) with density λ. In particular, each transmitter is assumed to follow uniformly independent identical distribution (i.i.d.). We consider a legitimate receiver, located in the center of this network. We assume there is an eavesdropper *E* with distance *D* away from the boundary of this communication area, trying to wiretap the confidential communications within the communication area. In order to protect the legitimate transmission, we place multiple friendly jammers at the circular boundary around the protected communication area.

We assume the channel experience Rayleigh fading and path loss. Therefore, the received power of a receiver with distance *r* from a transmitter is $hr^{-\alpha }$, where *h* is a random variable following an exponential distribution with mean 1 and α is the path loss factor.

### 2.2. Antennas

**Figure 2:**
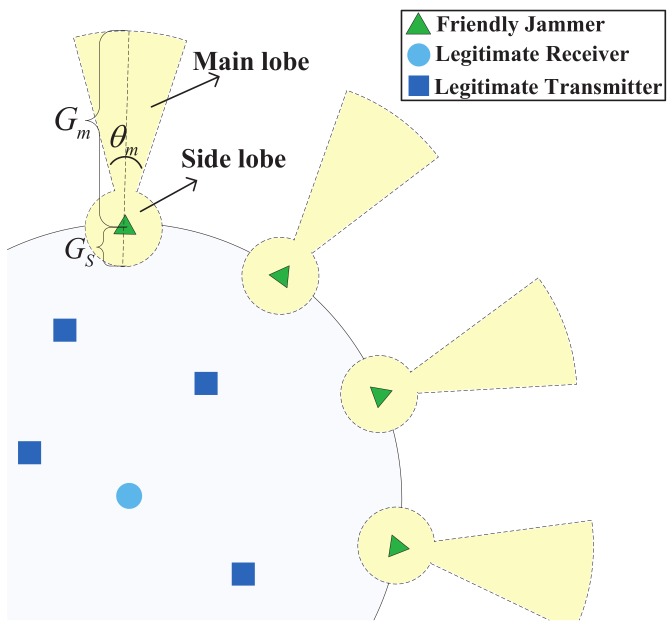
Friendly Jammers with Directional Antennas.

There are two types of antennas used in our network: an *omni-directional* antenna and a *directional antenna*. Omni-directional antennas radiate/collect radio signals into/from all directions equally. The antenna gain of omni-directional antenna is a constant in all directions, i.e., $G_{o}=1$. Different from an omni-directional antenna, a directional antenna can concentrate transmitting or receiving capability on some desired directions. Due to the high complexity to approximate a realistic directional antenna, we consider a simplified directional antenna model used in [24,25], as shown in Figure 2. This simplified directional antenna model consists of a main lobe $G_{m}$ within the beamwidth $\theta_{m}$ and a side lobe $G_{s}$ for all other directions. When $G_{m}$ and $\theta_{m}$ is given, $G_{s}$ can be calculated as follows [25],

(1) $$G_{s}=\frac{2-G_{m}\left(1-\cos\left(\frac{\theta_{m}}{2}\right)\right)}{1+\cos\frac{\theta_{m}}{2}}.$$

In this paper, the receiver, the transmitters and the eavedropper are assumed to be equipped with omni-directional antennas. Then, with respect to jammers, we consider two jammer strategies in this network: (i) OFJ scheme, in which jammers are equipped with omni-directional antennas; (ii) DFJ scheme, in which jammers equipped with directional antennas. For comparison purposes, we also consider a scheme: NFJ scheme, in which no friendly jammers are deployed.

## 3. Impacts of Jamming Schemes on Legitimate Transmission

In this section, we investigate the impacts of different schemes on the legitimate communications. In particular, we consider the probability of successful transmission as a metric to evaluate the transmission quality of legitimate communications. The probability of successful transmission is defined as follows,

**Definition** **1.** The probability of successful transmission is the expectation of the probability that a reference transmitter can successfully transmit with the legitimate receiver according to the distance between the receiver and the reference transmitter.

To guarantee the successful transmission of legitimate communication, the signal-to-interference- noise-ratio (SINR) at the legitimate receiver, denoted by ${SINR}_{T}$, must be no less than a threshold *T*. In particular, when we consider the communication between a reference transmitter $t_{0}$ and the receiver with distance $r_{0}$, ${SINR}_{T}$ can be expressed as

(2) $$SINR_{T}=\frac{G_{t}G_{r}P_{t}h_{0}{r_{0}}^{-\alpha }}{\sigma^{2}+I_{t}+I_{j}}=\frac{P_{t}h_{0}{r_{0}}^{-\alpha }}{\sigma^{2}+I_{t}+I_{j}},$$

where $P_{t}$ is the transmission power of transmitters, $G_{t}$ and $G_{r}$ are the antenna gains of the reference transmitter and the receiver, respectively (where we have $G_{t}=G_{r}=1$ since that the transmitters and receivers are equipped with omni-directional antennas), $I_{t}=\sum_{i\in \Phi /t_{0}}G_{t}G_{r}P_{t}h_{i}r_{i}^{-\alpha }=\sum_{i\in \Phi /t_{0}}P_{t}h_{i}r_{i}^{-\alpha }$ is the cumulative interference from legitimate users (where $r_{i}$ is the distance between the *i*th transmitter and the receiver), $I_{j}=\sum_{k=1}^{N}G_{j}G_{r}P_{j}h_{k}R^{-\alpha }=\sum_{k=1}^{N}G_{j}P_{j}h_{k}R^{-\alpha }$ is the cumulative interference from *N* jammers to the receiver (where $P_{j}$ is the transmission power of the jammers and $G_{j}$ is the antenna gain of the jammers), and $\sigma^{2}$ is the noise power.

Thus, we have the probability of successful transmission, denoted by $P_{T}$, as follows,

(3) $$P_{T}=\int_{0}^{R}P\left[{SINR}_{T}\geq T\left|r_{0}\right.\right]f_{r}\left(r_{0}\right)dr_{0}=\int_{0}^{R}P\left[\frac{P_{t}h_{0}{r_{0}}^{-\alpha }}{\sigma^{2}+I_{t}+I_{j}}\geq T\left|r_{0}\right.\right]f_{r}\left(r_{0}\right)dr_{0},$$

where $f_{r}\left(r_{0}\right)$ is the probability density function of the distance between the reference transmitter and the receiver $r_{0}$.

Then, we investigate the probability of successful transmission $P_{T}$ according to the three jammer strategies: NFJ, OFJ, and DFJ schemes.

### 3.1. Impact of NFJ Scheme

In NFJ scheme, there is no friendly jammer at the boundary of the communication area. Therefore, the cumulative interference from friendly jammers $I_{j}=0$. Then we have $P_{T}$ in the NFJ scheme as in the following theorem.

**Theorem** **1.** *In the NFJ scheme, the probability of successful transmission is*

(4) $$P_{T}=\frac{2^{M}}{R^{2M}}\int_{0}^{R}\exp\left(-T_{p}{r_{0}}^{\alpha }\sigma^{2}\right){\left(\int_{0}^{R}\frac{r^{1+\alpha }}{r^{\alpha }+T{r_{0}}^{\alpha }}dr\right)}^{M-1}dr_{0},$$

*where $T_{p}=T/P_{t}$, and M is the expectation of the number of legitimate transmitters in the communication area.*

**Proof.**  Let the distance between the receiver and a transmitter be *r*. Since the receiver is located at the center of the circular area and each transmitter follows uniformly i.i.d., the probability density function of *r* is as follows,

(5) $$f_{r}\left(r\right)=\frac{2\pi r}{\pi R^{2}}=\frac{2r}{R^{2}},0<r\leq R.$$

After combining Equations (5) and (2), we have $P_{T}$ as follows,

(6) $$\begin{array}{cc} P_{T} & =\int_{0}^{R}P\left[\frac{P_{t}h_{0}{r_{0}}^{-\alpha }}{\sigma^{2}+I_{t}}\geq T|r_{0}\right]f_{r}\left(r_{0}\right)dr_{0}\\ & =\int_{0}^{R}P\left[h_{0}\geq T_{p}{r_{0}}^{\alpha }\left(\sigma^{2}+I_{t}\right)|r_{0}\right]2r_{0}R^{-2}dr_{0}, \end{array}$$

where $T_{p}=T/P_{t}$.

Since *h* is a random variable following an exponential distribution with mean 1, $P[h_{0}\geq T_{p}r_{0}^{\alpha }\left(\sigma^{2}+I_{t}\right)|r_{0}]$ in Equation (6) can be expressed as

(7) $$\begin{array}{cc} P & \left[h_{0}\geq T_{p}r_{0}^{\alpha }\left(\sigma^{2}+I_{t}\right)|r_{0}\right]\\ & =E_{I_{t}}\left[\exp\left(\left(-T_{p}{r_{0}}^{\alpha }\right)\left(\sigma^{2}+I_{t}\right)\right)|r_{0}\right]\\ & =e^{-T_{p}{r_{0}}^{\alpha }\sigma^{2}}E_{I_{t}}\left[\exp\left(-T_{p}{r_{0}}^{\alpha }I_{t}\right)\right]. \end{array}$$

Next, we calculate $E_{I_{t}}\left[\exp\left(-T_{p}{r_{0}}^{\alpha }I_{t}\right)\right]$. If we denote the expected number of legitimate transmitters by *M*, we can derive the expression of $E_{I_{t}}\left[\exp\left(-T_{p}{r_{0}}^{\alpha }I_{t}\right)\right]$ as follows,

(8) $$\begin{array}{cc} E_{I_{t}}\left[\exp\left(-T_{p}{r_{0}}^{\alpha }I_{t}\right)\right]= & E_{\Phi ,\{h_{i}\}}\left[\exp\left(-T_{p}{r_{0}}^{\alpha }\sum_{i\in \Phi /t_{0}}P_{t}h_{i}r_{i}^{-\alpha }\right)\right]\\ = & E_{\left\{r_{i}\right\},\left\{h_{i}\right\}}\left[\exp\left(-T_{p}P_{t}{r_{0}}^{\alpha }\sum_{i=1}^{M-1}h_{i}r_{i}^{-\alpha }\right)\right]\\ = & E_{\left\{r_{i}\right\},\left\{h_{i}\right\}}\left[\prod_{i=1}^{M-1}\exp\left(-T{r_{0}}^{\alpha }h_{i}r_{i}^{-\alpha }\right)\right]\\ \overset{\left(a\right)}{=} & {\left[E_{r,h}\left(\exp\left(-T{r_{0}}^{\alpha }hr^{-\alpha }\right)\right)\right]}^{M-1}\\ \overset{\left(b\right)}{=} & {\left[E_{r}\left(\frac{1}{1+T{r_{0}}^{\alpha }r^{-\alpha }}\right)\right]}^{M-1}\\ = & {\left[\int_{0}^{R}(\frac{1}{1+T{\left(r_{0}/r\right)}^{\alpha }})f_{r}\left(r\right)dr\right]}^{M-1}\\ = & {\left[\frac{2}{R^{2}}\int_{0}^{R}(\frac{r^{1+\alpha }}{r^{\alpha }+T{r_{0}}^{\alpha }})dr\right]}^{M-1}, \end{array}$$

where (a) is derived from the assumption that Rayleigh fading factor of each channel follows exponentially i.i.d., and (b) can be derived from the property of moment generating function of exponential variable.

Substituting Equation (8) into the corresponding part of Equation (7), we have

(9) $$P\left[h_{0}\geq T_{P}r^{\alpha }\left(\sigma^{2}+I_{t}\right)\right]=\exp\left(-T_{p}{r_{0}}^{\alpha }\sigma^{2}\right)\cdot {\left(\frac{2}{R^{2}}\int_{0}^{R}\frac{r^{1+\alpha }}{r^{\alpha }+T\cdot {r_{0}}^{\alpha }}dr\right)}^{M-1}.$$

After plugging Equation (9) into Equation (6), we can obtain $P_{T}$ in Theorem 1. ☐

### 3.2. Impact of OFJ Scheme

In the OFJ scheme, the friendly jammers placed at the boundary of communication area are equipped with omni-directional antennas, i.e., the antenna gain of jammers is $G_{j}=G_{o}=1$. Therefore, $I_{j}$ in Equation (3) can be expressed as $\sum_{k=1}^{N}P_{j}h_{k}R^{-\alpha }$. Then we give $P_{T}$ in the OFJ scheme by the following theorem.

**Theorem** **2.** *In the OFJ Scheme, the probability of successful transmission is*

(10) $$P_{T}=\frac{2^{M}}{R^{2M}}\int_{0}^{R}\exp\left(-T_{p}{r_{0}}^{\alpha }\sigma^{2}\right){\left[1+T_{p}{\left(r_{0}R\right)}^{-\alpha }P_{j}\right]}^{-N}{\left(\int_{0}^{R}\frac{r^{1+\alpha }}{r^{\alpha }+T{r_{0}}^{\alpha }}dr\right)}^{M-1}dr_{0}.$$

**Proof.**  Similar to the proof of Theorem 1, the probability of successful transmission $P_{T}$ in the OFJ scheme can be expressed as follows,

(11) $$P_{T}=\int_{0}^{R}P\left[h_{0}\geq T_{p}{r_{0}}^{\alpha }\left(\sigma^{2}+I_{t}+I_{j}\right)|r_{0}\right]2r_{0}R^{-2}dr_{0},$$

where $P[h_{0}\geq T_{p}{r_{0}}^{\alpha }\left(\sigma^{2}+I_{t}+I_{j}\right)|r_{0}]$ can be derived as follows,

(12) $$\begin{array}{cc} P & \left[h_{0}\geq T_{p}r^{\alpha }\left(\sigma^{2}+I_{t}+I_{j}\right)|r_{0}\right]\\ & =E_{I_{t},I_{j}}\left[\exp\left(\left(-T_{p}{r_{0}}^{\alpha }\right)\left(\sigma^{2}+I_{t}+I_{j}\right)\right)|r_{0}\right]\\ & =e^{-T_{p}{r_{0}}^{\alpha }\sigma^{2}}E_{I_{t}}\left[\exp\left(-T_{p}{r_{0}}^{\alpha }I_{t}\right)\right]E_{I_{j}}\left[\exp\left(-T_{p}{r_{0}}^{\alpha }I_{j}\right)\right], \end{array}$$

where $E_{I_{t}}\left[\exp\left(-T_{p}{r_{0}}^{\alpha }I_{t}\right)\right]$ is given by Equation (8), and $E_{I_{j}}\left[\exp\left(-T_{p}{r_{0}}^{\alpha }I_{j}\right)\right]$ can be calculated by

(13) $$\begin{array}{cc} E_{I_{j}}\left[\exp\left(-T_{p}{r_{0}}^{\alpha }I_{j}\right)\right]= & E_{h}\left[\exp\left(-T_{p}{r_{0}}^{-\alpha }\sum_{n=1}^{N}P_{j}h_{n}R^{-\alpha }\right)\right]\\ = & E_{h}\left[\prod_{n=1}^{N}\exp(-T_{p}{r_{0}}^{-\alpha }P_{j}R^{-\alpha }h_{n})\right]\\ = & \prod_{n=1}^{N}E_{h}\left[\exp\left(-T_{p}{r_{0}}^{-\alpha }P_{j}R^{-\alpha }h_{n}\right)\right]\\ = & {\left[1+T_{p}{\left(r_{0}R\right)}^{-\alpha }P_{j}\right]}^{-N}. \end{array}$$

Substituting Equations (8) and (13) into the corresponding parts of Equation (12), we have

(14) $$P\left[h_{0}\geq T_{P}r^{\alpha }\left(\sigma^{2}+I_{t}+I_{j}\right)\right]=\exp\left(-T_{p}{r_{0}}^{\alpha }\sigma^{2}\right)\cdot {\left[1+T_{p}{\left(r_{0}R\right)}^{-\alpha }P_{j}\right]}^{-N}\cdot {\left(\frac{2}{R^{2}}\int_{0}^{R}\frac{r^{1+\alpha }}{r^{\alpha }+T\cdot {r_{0}}^{\alpha }}dr\right)}^{M-1}.$$

By plugging Equation (14) into Equation (11), we can obtain $P_{T}$ of OFJ scheme in Theorem 2. ☐

### 3.3. Impact of DFJ Scheme

In DFJ scheme, the jammers placed at the boundary are equipped with directional antennas. In particular, we can find that the receiver can be only affected by the side lobe of directional antennas, as shown in Figure 2. Therefore, we have $G_{j}$ =$G_{s}$. Thus, $I_{j}$ in Equation (3) can be expressed as $\sum_{k=1}^{N}G_{s}P_{j}h_{k}R^{-\alpha }$. Then, we obtain $P_{T}$ in the DFJ scheme by the following theorem.

**Theorem** **3.** *In the DFJ scheme, the probability of successful transmission is*

(15) $$P_{T}=\frac{2^{M}}{R^{2M}}\int_{0}^{R}\exp\left(-T_{p}{r_{0}}^{\alpha }\sigma^{2}\right){\left[1+T_{p}{\left(r_{0}R\right)}^{-\alpha }P_{j}G_{s}\right]}^{-N}{\left(\int_{0}^{R}\frac{r^{1+\alpha }}{r^{\alpha }+T{r_{0}}^{\alpha }}dr\right)}^{M-1}dr_{0}.$$

**Proof.**  Similar to the proof of Theorems 1 and 2, $P_{T}$ in DFJ can be expressed as

(16) $$\begin{array}{cc} P_{T}= & \int_{0}^{R}P\left[\frac{P_{t}h_{0}{r_{0}}^{-\alpha }}{\sigma^{2}+I_{t}+I_{j}}\geq T|r_{0}\right]f_{r}\left(r_{0}\right)dr_{0}\\ = & \int_{0}^{R}E_{I_{t}}\left[\exp\left(-T_{p}{r_{0}}^{\alpha }I_{t}\right)\right]E_{I_{j}}\left[\exp\left(-T_{p}{r_{0}}^{\alpha }I_{j}\right)\right]\cdot 2r_{0}R^{-2}e^{-T_{p}{r_{0}}^{\alpha }\sigma^{2}}dr_{0}, \end{array}$$

where $E_{I_{t}}\left[\exp\left(-T_{p}{r_{0}}^{\alpha }I_{t}\right)\right]$ is given by Equation (8), and $E_{I_{j}}\left[\exp\left(-T_{p}{r_{0}}^{\alpha }I_{j}\right)\right]$ can be calculated by

(17) $$\begin{array}{cc} E_{I_{j}}\left[\exp\left(-T_{p}{r_{0}}^{\alpha }I_{j}\right)\right]= & E_{h}\left[\exp\left(-T_{p}{r_{0}}^{-\alpha }\sum_{n=1}^{N}P_{j}G_{s}h_{n}R^{-\alpha }\right)\right]\\ = & \prod_{n=1}^{N}E_{h}\left[\exp\left(-T_{p}{r_{0}}^{-\alpha }P_{j}G_{s}R^{-\alpha }h_{n}\right)\right]\\ = & {\left[1+T_{p}{\left(r_{0}R\right)}^{-\alpha }P_{j}G_{s}\right]}^{-N}. \end{array}$$

After plugging Equations (8) and (17) into Equation (16), we can obtain $P_{T}$ of DFJ scheme in Theorem 3. ☐

## 4. Analysis on Probability of Eavesdropping Attacks

In this section, we analyze the influence of friendly jammers on the probability of an eavesdropping attack of this network. In particular, we use the probability of an eavesdropping attack as the metric to evaluate the possibility of being eavesdropped on in this network. We assume that if the eavesdropper can wiretap any of the transmitters, this network can be seen as being attacked. Based on this assumption, we give the definition of the probability of eavesdropping attack as follows.

**Definition** **2.** The probability of an eavesdropping attack is the probability that the eavesdropper can wiretap any of the transmitters.

Before we analyze the probability of an eavesdropping attack, we first analyze the probability that a certain transmitter can be wiretapped by the eavesdropper, denoted by $P_{e}$. If the eavesdropper can wiretap a transmitter $t_{0}$ with distance $l_{0}$, the SINR at the eavesdropper, denoted by $SINR_{E}$, has to be no less than a threshold β. Thus, $P_{e}$ can be expressed as follows,

(18) $$\begin{array}{cc} P_{e}=E_{l_{0}}\left[P\left(SINR_{E}\geq \beta |l_{0}\right)\right]= & \int_{D}^{D+2R}P\left[\frac{G_{t}G_{e}P_{t}h_{0}{l_{0}}^{-\alpha }}{\sigma^{2}+I_{te}+I_{je}}\geq \beta |l_{0}\right]f_{l}\left(l_{0}\right)dl_{0}\\ = & \int_{D}^{D+2R}P\left[\frac{P_{t}h_{0}{l_{0}}^{-\alpha }}{\sigma^{2}+I_{te}+I_{je}}\geq \beta |l_{0}\right]f_{l}\left(l_{0}\right)dl_{0} \end{array}$$

where $G_{e}$ is the antenna gain of the eavesdropper (we have $G_{e}=1$ since the eavesdropper is equipped by an omni-directional antenna), $I_{te}=\sum_{i\in \Phi /t_{0}}G_{t}G_{e}P_{t}h_{i}l_{i}^{-\alpha }=\sum_{i\in \Phi /t_{0}}P_{t}h_{i}l_{i}^{-\alpha }$ is the cumulative interference from transmitters to the eavesdropper (where $l_{i}$ is the distance between the *i*th transmitter and the eavesdropper), $I_{je}$ is the cumulative interference from the jammers to the eavesdropper, which will be elaborated later according to different jammer schemes, and $f_{l}\left(l_{0}\right)$ is the probability density function of $l_{0}$.

Next, based on the analysis of $P_{e}$, we give the probability of eavesdropping attack, denoted by $P_{E}$, as follows,

(19) $$P_{E}=1-{\left(1-P_{e}\right)}^{M}.$$

The impact of friendly jammers on the eavesdropping attacks will then be investigated. In particular, we will derive the probability of an eavesdropping attack $P_{E}$ of an eavesdropper in the NFJ scheme, OFJ scheme and DFJ scheme as follows, respectively.

### 4.1. Impact of NFJ Scheme

Firstly we consider the NFJ scheme in which there is no friendly jammer on the boundary of communication area. In this case, the interference from friendly jammers to eavesdropper $I_{je}=0$, then we have the following theorem:

**Theorem** **4.** *In the NFJ scheme, the probability of eavesdropping attack $P_{E}$ is*

(20) $$P_{E}\;=\;1\;-\;{\left(1\;-\;{\left(\frac{2}{\pi R^{2}}\right)}^{M}\cdot \int_{D}^{D+2R}\exp\left(-\beta_{p}{l_{0}}^{\alpha }\sigma^{2}\right)l_{0}{Z(l_{0})}^{M-1}\cdot \arccos\left[\frac{D}{l_{0}}\;+\;\frac{{l_{0}}^{2}-D^{2}}{2l_{0}\left(R\;+\;D\right)}\right]dl_{0}\right)}^{M},$$

*where $\beta_{p}=\frac{\beta }{P_{t}}$, $V\left(l_{0}\right)=\beta_{p}{l_{0}}^{\alpha }P_{j}$ and*

$$Z\left(l_{0}\right)=\int_{D}^{D+2R}(\frac{l^{\alpha +1}}{l^{\alpha }+\beta {l_{0}}^{\alpha }})\arccos\left[\frac{D}{l}+\frac{l^{2}-D^{2}}{2l(R+D)}\right]dl.$$

**Proof.**  We denote the distance between the eavesdropper and a transmitter by *l*. The probability density function of *l* can be expressed as follows [26],

(21) $$f_{l}\left(l\right)=\frac{2l}{\pi R^{2}}\arccos\left[\frac{D}{l}+\frac{l^{2}-D^{2}}{2l(R+D)}\right],D\leq l\leq D+2R.$$

Then $P_{e}$ can be expressed as

(22) $$P_{e}=\int_{D}^{D+2R}\frac{2l_{0}}{\pi R^{2}}P[h_{0}\geq \beta_{p}{l_{0}}^{\alpha }\left(\sigma^{2}+I_{te}\right)|l_{0}]\cdot \arccos\left(\frac{d}{l_{0}}+\frac{{l_{0}}^{2}-d^{2}}{2l_{0}\left(R+d\right)}\right)dl_{0},$$

where $\beta_{p}=\frac{\beta }{P_{t}}$.

Since $h_{0}$ is a random variable following an exponential distribution with mean 1, $P[h_{0}\geq \beta_{p}{l_{0}}^{\alpha }\left(\sigma^{2}+I_{te}\right)|l_{0}]$ can be expressed as

(23) $$\begin{array}{cc} P & \left[h_{0}\geq \beta_{p}{l_{0}}^{\alpha }\left(\sigma^{2}+I_{te}\right)|l_{0}\right]\\ & =E_{I_{te}}\left[P\left(h_{0}\geq \beta_{p}{l_{0}}^{\alpha }\left(\sigma^{2}+I_{te}\right)|l_{0}\right)\right]\\ & =E_{I_{te}}\left[\exp\left(\left(-\beta_{p}{l_{0}}^{\alpha }\right)\left(\sigma^{2}+I_{te}\right)\right)|l_{0}\right]\\ & =e^{-\beta_{p}{l_{0}}^{\alpha }\sigma^{2}}\cdot E_{I_{te}}\left[\exp\left(-\beta_{p}{l_{0}}^{\alpha }I_{te}\right)\right]. \end{array}$$

Following the similar approach in deriving Equation (8), the expression of $E_{I_{te}}\left[\exp\left(-\beta_{p}l^{\alpha }I_{te}\right)\right]$ can be derived by the following equation,

(24) $$\begin{array}{cc} & E_{I_{te}}\left[\exp\left(-\beta_{p}{l_{0}}^{\alpha }I_{te}\right)\right]\\ & \;\;=\;E_{\Phi ,\{h_{i}\}}\left[\exp\left(-\beta_{p}{l_{0}}^{\alpha }\sum_{i\in \Phi /b_{0}}P_{t}h_{i}l_{i}^{-\alpha }\right)\right]\\ & \;\;=\;{\left[\int_{D}^{D+2R}(\frac{1}{1+\beta {\left(l_{0}/l\right)}^{\alpha }})f_{l}\left(l\right)dl\right]}^{M-1}\\ & \;\;=\;{\left[\frac{2}{\pi R^{2}}\int_{D}^{D+2R}(\frac{l^{\alpha +1}}{l^{\alpha }\;+\;\beta {l_{0}}^{\alpha }})\arccos\left[\frac{D}{l}\;+\;\frac{l^{2}-D^{2}}{2l(R\;+\;D)}\right]dl\right]}^{M\;-\;1}. \end{array}$$

If we set $Z\left(l_{0}\right)=\int_{D}^{D+2R}(\frac{l^{\alpha +1}}{l^{\alpha }+\beta {l_{0}}^{\alpha }})\arccos\left[\frac{D}{l}+\frac{l^{2}-D^{2}}{2l(R+D)}\right]dl$, Equation (24) can be expressed as

(25) $$E_{I_{te}}\left[\exp\left(-\beta_{p}{l_{0}}^{\alpha }I_{te}\right)\right]={\left[\frac{2Z(l_{0})}{\pi R^{2}}\right]}^{M-1}.$$

After plugging Equation (25) into the Equation (23), and substituting the new expression of Equation (23) into Equation (22), we obtain the result of $P_{e}$ as follows,

(26) $$P_{e}={\left(\frac{2}{\pi R^{2}}\right)}^{M}\cdot \int_{D}^{D+2R}\exp\left(-\beta_{p}{l_{0}}^{\alpha }\sigma^{2}\right)l_{0}{Z(l_{0})}^{M-1}\cdot \arccos\left[\frac{D}{l_{0}}+\frac{{l_{0}}^{2}-D^{2}}{2l_{0}\left(R+D\right)}\right]dl_{0}.$$

Substituting $P_{e}$ in Equation (26) into Equation (19), we derive the probability of eavesdropping attack $P_{E}$ of the NFJ scheme as given in Theorem 4. ☐

### 4.2. Impact of OFJ Scheme

Then we investigate the OFJ scheme, where friendly jammers are equipped with omni-directional antennas. In order to derive $P_{e}$, we need to calculate the interference from jammers to eavesdropper $I_{je}$ first.

**Figure 3:**
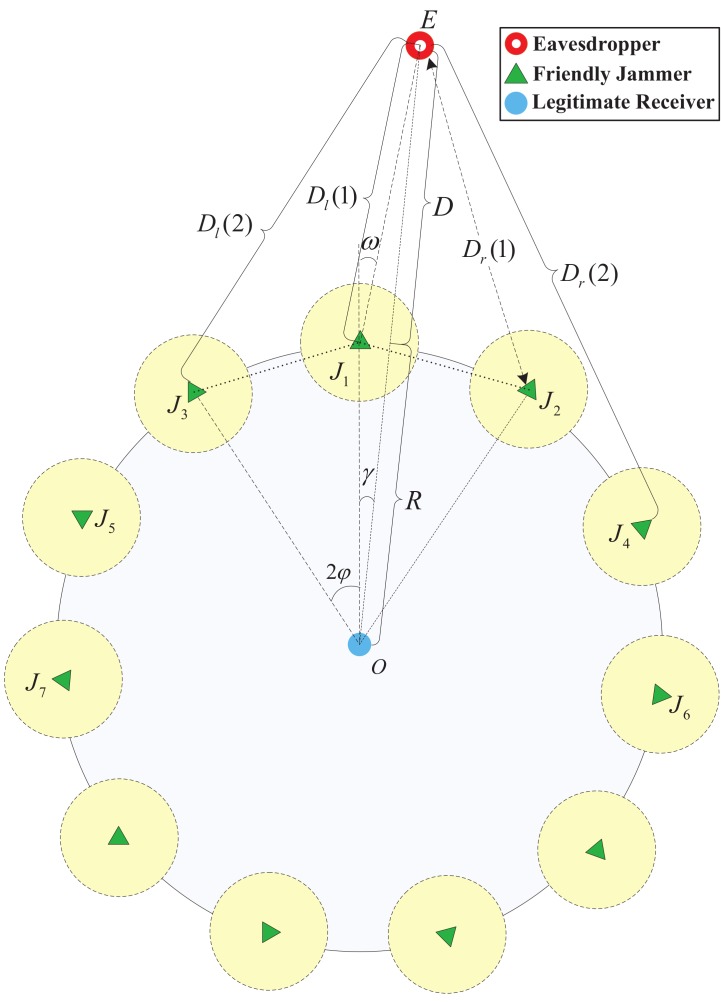
Geometrical relationship of the friendly jammers and the eavesdropper.

Figure 3 shows the geometrical relationships of the friendly jammers and the eavesdropper. Without loss of generality, we label the jammer which is nearest to the eavesdropper as $J_{1}$ and the jammer $J_{1}$ is at the left-hand-side of the eavesdropper. Then we label $J_{2m}$ (where m=1,2,3,…) as *m*th nearest jammer at the right-hand-side of jammer $J_{1}$ separately. Similarly, we label $J_{2n+1}$ (where n=1,2,3,…) as the *n*th nearest jammer at the left-hand-side of jammer $J_{1}$ separately.

From the observation point *O*, 2φ is the relative degree between neighbour jammers and γ is the degree between jammer $J_{1}$ and eavesdropper *E*. Since the number of jammers is *N*, we have $2\varphi =\frac{2\pi }{N}$. Due to the fact that the eavesdropper is randomly located outside of the protected communication area, we have the probability density function of variable γ,

(27) $$f_{\gamma }\left(\gamma \right)=\frac{1}{\varphi }\;,0\leq \gamma \leq \varphi .$$

Then we can calculate the cumulative interference of the jammers to the eavesdropper based on their geometrical relationships, which is given by the following lemma.

**Lemma** **1.** *When the friendly jammers are equipped with omni-directional antennas, the cumulative interference from the jammers to the eavesdropper is*

(28) $$I_{je}=\left\{\begin{array}{c} P_{j}\left[\sum_{x=-\frac{N-1}{2}}^{\frac{N-1}{2}}h_{x}D_{je}{\left(x\right)}^{-\alpha }\right],\;N\;\mathrm{is}\;\mathrm{odd}\\ P_{j}\left[\sum_{x=-\frac{N}{2}}^{\frac{N-2}{2}}h_{x}D_{je}{\left(x\right)}^{-\alpha }\right],\;N\;\mathrm{is}\;\mathrm{even} \end{array}\right.,$$

*where* $D_{je}\left(x\right)=\sqrt{R^{2}+L^{2}-2RL\cos\left(2x\varphi +\gamma \right)}$ *.*

**Proof.**  We present the proof of Lemma 1 in Appendix A.

With the interference from the jammers to the eavesdropper $I_{je}$, we obtain the probability of eavesdropping attack $P_{E}$ as the following theorem.

**Theorem** **5.** *In the OFJ scheme, the probability of an eavesdropping attack $P_{E}$ of an eavesdropper is:*

(29) $$P_{E}\;=\;1\;-\;{\left(1\;-\;{\left(\frac{2}{\pi R^{2}}\right)}^{M}\int_{D}^{D+2R}\exp\left(-\beta_{p}{l_{0}}^{\alpha }\sigma^{2}\right)l_{0}{Z(l_{0})}^{M-1}W\left(l_{0}\right)\arccos\left[\frac{D}{l_{0}}\;+\;\frac{{l_{0}}^{2}\;-\;D^{2}}{2l_{0}\left(R\;+\;D\right)}\right]dl_{0}\right)}^{M},$$

*where*

$$Z\left(l_{0}\right)=\int_{D}^{D+2R}(\frac{l^{\alpha +1}}{l^{\alpha }+\beta {l_{0}}^{\alpha }})\arccos\left[\frac{D}{l}+\frac{l^{2}-D^{2}}{2l(R+D)}\right]dl,$$

*and*

$$\begin{array}{c} W\left(l_{0}\right)=\left\{\begin{array}{c} \prod_{x=-\;\frac{N\;-\;1}{2}}^{\frac{N\;-\;1}{2}}\int_{0}^{\varphi }\frac{1}{\varphi (1\;+\;V\left(l_{0}\right)D_{je}{\left(x\right)}^{\;-\;\alpha })}d\gamma ,\;N\;\mathrm{is}\;\mathrm{odd}\\ \prod_{x=-\;\frac{N}{2}}^{\frac{N\;-\;2}{2}}\int_{0}^{\varphi }\frac{1}{\varphi (1\;+\;V\left(l_{0}\right)D_{je}{\left(x\right)}^{\;-\;\alpha })}d\gamma ,\;N\;\mathrm{is}\;\mathrm{even} \end{array}\right., \end{array}$$

*in which $V\left(l_{0}\right)=\beta_{p}{l_{0}}^{\alpha }P_{j}$ and $\beta_{p}=\frac{\beta }{P_{t}}$.*

**Proof.**  Following the similar approach to the proof of Theorem 4, we can get the probability that a certain transmitter can be tapped denoted by $P_{e}$ as follows,

(30) $$\begin{array}{cc} P_{e}= & E_{l_{0}}\left[P\left(SINR_{E}\geq \beta |l_{0}\right)\right]\\ = & \int_{D}^{D+2R}P\left[\frac{P_{t}h_{0}{l_{0}}^{-\alpha }}{\sigma^{2}+I_{te}+I_{je}}\geq \beta |l_{0}\right]f_{l}\left(l_{0}\right)dl_{0}\\ = & \int_{D}^{D+2R}\frac{2l_{0}}{\pi R^{2}}P[h_{0}\geq \beta_{p}{l_{0}}^{\alpha }\left(\sigma^{2}+I_{te}+I_{je}\right)|l_{0}]\cdot \arccos\left(\frac{d}{l_{0}}+\frac{{l_{0}}^{2}-d^{2}}{2l_{0}\left(R+d\right)}\right)dl_{0}, \end{array}$$

where $\beta_{p}=\frac{\beta }{P_{t}}$.

Since $h_{0}$ is a random variable following an exponential distribution with mean 1, $P[h_{0}\geq \beta_{p}{l_{0}}^{\alpha }\left(\sigma^{2}+I_{te}+I_{je}\right)|l_{0}]$ can be expressed as

(31) $$P\left[h_{0}\geq \beta_{p}{l_{0}}^{\alpha }\left(\sigma^{2}+I_{te}+I_{je}\right)|l_{0}\right]=e^{-\beta_{p}{l_{0}}^{\alpha }\sigma^{2}}\cdot E_{I_{te}}\left[\exp\left(-\beta_{p}{l_{0}}^{\alpha }I_{te}\right)\right]\cdot E_{I_{je}}\left[\exp\left(-\beta_{p}{l_{0}}^{\alpha }I_{je}\right)\right],$$

where $E_{I_{te}}\left[\exp\left(-\beta_{p}{l_{0}}^{\alpha }I_{te}\right)\right]={\left[\frac{2Z(l_{0})}{\pi R^{2}}\right]}^{M-1}$ given by Equation (24).

Then we calculate $E_{I_{je}}\left[\exp\left(-\beta_{p}{l_{0}}^{\alpha }I_{je}\right)\right]$. For simplicity, we denote $W\left(l_{0}\right)=E_{I_{je}}\left[\exp\left(-\beta_{p}{l_{0}}^{\alpha }I_{je}\right)\right]$, $V\left(l_{0}\right)=\beta_{p}{l_{0}}^{\alpha }P_{j}$. With the expression of $I_{je}$ given in Lemma 1, we can obtain $W(l_{0})$ given by the following equation,

(32) $$\begin{array}{c} \begin{array}{c} W\left(l_{0}\right)=E_{I_{je}}\left[\exp\left(-\beta_{p}{l_{0}}^{\alpha }I_{je}\right)\right]\\ =\left\{\begin{array}{c} E_{h,\gamma }\left[\exp\left(-\sum_{x=-\;\frac{N\;-\;1}{2}}^{\frac{N\;-\;1}{2}}V\left(l_{0}\right)h_{k}D_{je}{\left(x\right)}^{\;-\;\alpha }\right)\right],\;N\;\mathrm{is}\;\mathrm{odd};\\ E_{h,\gamma }\left[\exp\left(-\sum_{x=-\;\frac{N}{2}}^{\frac{N\;-\;2}{2}}V\left(l_{0}\right)h_{k}D_{je}{\left(x\right)}^{\;-\;\alpha }\right)\right],\;N\;\mathrm{is}\;\mathrm{even}. \end{array}\right. \end{array}\;\overset{\left(c\right)}{=}\left\{\begin{array}{c} E_{\gamma }\left[\prod_{x=-\;\frac{N\;-\;1}{2}}^{\frac{N\;-\;1}{2}}E_{h}\left[\exp\left(-V\left(l_{0}\right)D_{je}{\left(x\right)}^{\;-\;\alpha }h_{k}\right)\right]\right],\;N\;\mathrm{is}\;\mathrm{odd};\\ E_{\gamma }\left[\prod_{x=-\;\frac{N}{2}}^{\frac{N\;-\;2}{2}}E_{h}\left[\exp\left(-V\left(l_{0}\right)D_{je}{\left(x\right)}^{\;-\;\alpha }h_{k}\right)\right]\right],\;N\;\mathrm{is}\;\mathrm{even}. \end{array}\right. \end{array}\;\overset{\left(d\right)}{=}\left\{\begin{array}{c} \prod_{x=-\;\frac{N\;-\;1}{2}}^{\frac{N\;-\;1}{2}}E_{\gamma }\left[\frac{1}{1\;+\;V\left(l_{0}\right)D_{je}{\left(x\right)}^{\;-\;\alpha }}\right],\;N\;\mathrm{is}\;\mathrm{odd};\\ \prod_{x=-\;\frac{N}{2}}^{\frac{N\;-\;2}{2}}E_{\gamma }\left[\frac{1}{1\;+\;V\left(l_{0}\right)D_{je}{\left(x\right)}^{\;-\;\alpha }}\right],\;N\;\mathrm{is}\;\mathrm{even}. \end{array}\right.\;\overset{\left(e\right)}{=}\left\{\begin{array}{c} \prod_{x=-\;\frac{N\;-\;1}{2}}^{\frac{N\;-\;1}{2}}\int_{0}^{\varphi }\frac{1}{\varphi (1\;+\;V\left(l_{0}\right)D_{je}{\left(x\right)}^{\;-\;\alpha })}d\gamma ,\;N\;\mathrm{is}\;\mathrm{odd};\\ \prod_{x=-\;\frac{N}{2}}^{\frac{N\;-\;2}{2}}\int_{0}^{\varphi }\frac{1}{\varphi (1\;+\;V\left(l_{0}\right)D_{je}{\left(x\right)}^{\;-\;\alpha })}d\gamma ,\;N\;\mathrm{is}\;\mathrm{even}. \end{array}\right..$$

where (c) is derived from the independence between an eavesdropper’s location and the distribution of fading channel, (d) follows from the property of moment generating function of exponential variable, (e) is derived with the probability density function of γ as given in Equation (27).

After plugging Equation (32) into Equation (31), and substituting the new expression of Equation (31) into Equation (30), we obtain the result of $P_{e}$ as given in the following expression,

(33) $$\begin{array}{cc} P_{e}= & {\left(\frac{2}{\pi R^{2}}\right)}^{M}\cdot \int_{D}^{D+2R}\exp\left(-\beta_{p}{l_{0}}^{\alpha }\sigma^{2}\right)l_{0}{Z(l_{0})}^{M-1}W\left(l_{0}\right)\arccos\left[\frac{D}{l_{0}}+\frac{{l_{0}}^{2}-D^{2}}{2l_{0}\left(R+D\right)}\right]dl_{0}, \end{array}$$

Substituting the $P_{e}$ in Equation (33) into Equation (19), we obtain the result of $P_{E}$ given in Theorem 5. ☐

### 4.3. Impact of DFJ Scheme

In order to derive the probability of an eavesdropping attack $P_{E}$ in the DJF scheme, we need to evaluate the interference from the friendly jammers equipped with directional antenna to the eavesdropper.

**Figure 4:**
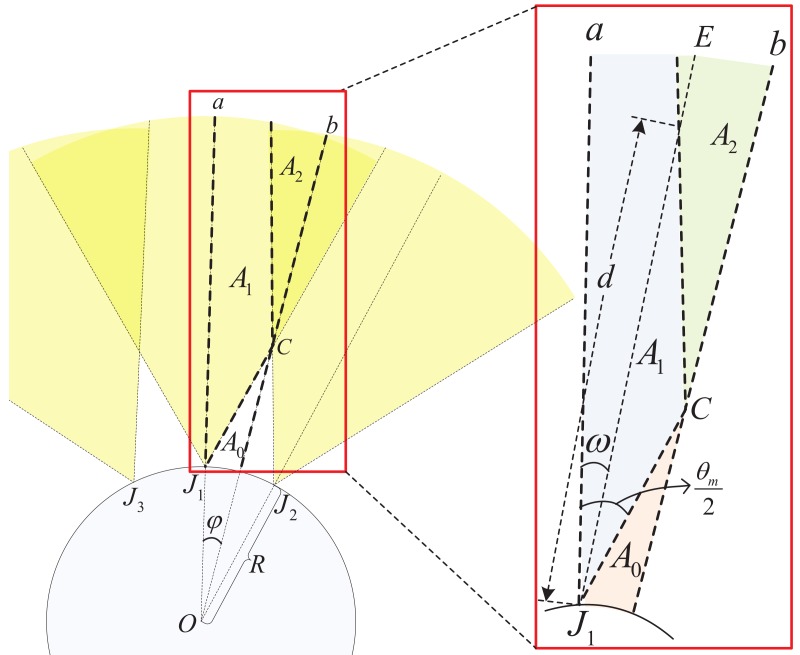
Geometrical relationship of friendly jammers (three jammers are shown).

However, when the jammers are deployed densely or the distance between the eavesdropper and the commmunication area *D* is large, there may be more than one jammer that interferes with the eavesdropper via their main lobes simultaneously (as shown in Figure 4). Therefore, we first investigate the number of friendly jammers which interfere with the eavesdropper via main lobes.

In Figure 4, we show the main lobes of 3 jammers. Due to the fact that the eavesdropper is nearest to the jammer $J_{1}$, the eavesdropper locates in the area between line *a* and line *b*, where line *a* is the extended line of $OJ_{1}$ and line *b* is the perpendicular bisector of segment $J_{1}J_{2}$.

The term of ω in Figure 4 is the degree between line *a* and $J_{1}E$. When $\omega \geq \frac{\theta_{m}}{2}$, the eavesdropper locates in area $A_{0}$, there is no jammers interfering it with its main lobe. When $\omega \leq \frac{\theta_{m}}{2}$, the area that the eavesdropper locates depends on the distance *D*. When $\omega \leq \frac{\theta_{m}}{2}$, the eavesdropper locates in $A_{1}$ if D≤d, there will be one jammer interfering with the eavesdropper via its main lobe; the eavesdropper locates in $A_{2}$ if D≥d, there will be two jammers interfering with the eavesdropper via their main lobes.

Similarly, we denote $A_{k}$ to be the intersection area of *k* jammers’ main lobe directions between line *a* and line *b*. When the eavesdropper locates in area $A_{k}$, there will be *k* jammers interfering with the eavesdropper via their main lobes. We denote the number of friendly jammers (interfering the eavesdropper via their main lobes) by $N_{d}$. When $N_{d}$ is fixed, the interference of directional jammers to eavesdroppers is shown in the following lemma.

**Lemma** **2.** *When $N_{d}=k$, the interference of jammers on eavesdropper in the DFJ scheme is*

(34) $$I_{je}\;=\;\left\{\begin{array}{c} P_{j}G_{n}\left[\sum_{x=-\frac{k-1}{2}}^{\frac{k-1}{2}}h_{x}D_{je}{\left(x\right)}^{-\alpha }\right]\;+\;I_{js},\;k\;\mathrm{is}\;\mathrm{odd}\\ P_{j}G_{n}\left[\sum_{x=-\frac{k}{2}}^{\frac{k-2}{2}}h_{x}D_{je}{\left(x\right)}^{-\alpha }\right]\;+\;I_{js},\;k\;\mathrm{is}\;\mathrm{even}\;and\;k\neq 0\\ \;I_{js}\;,\;k=0 \end{array}\right.,$$

*where $G_{n}=G_{m}-G_{s}$,* $D_{je}\left(x\right)=\sqrt{R^{2}+L^{2}-2RL\cos\left(2x\varphi +\gamma \right)}$ *, and*

$$I_{js}=\left\{\begin{array}{c} P_{j}G_{s}\left[\sum_{x=-\frac{N-1}{2}}^{\frac{N-1}{2}}h_{x}D_{je}{\left(x\right)}^{-\alpha }\right],\;N\;\mathrm{is}\;\mathrm{odd};\\ P_{j}G_{s}\left[\sum_{x=-\frac{N}{2}}^{\frac{N-2}{2}}h_{x}D_{je}{\left(x\right)}^{-\alpha }\right],\;N\;\mathrm{is}\;\mathrm{even}. \end{array}\right.$$

**Proof.**  We present the proof of Lemma 2 in Appendix B. ☐

With the interference from the jammers to the eavesdropper, we obtain the probability of eavesdropping attack $P_{E}$ as the following theorem,

**Theorem** **6.** *In DFJ scheme, the upper bound of probability of eavesdropping attack $P_{E}$ is given by*

$$\begin{array}{c} P_{E}=\frac{\varphi -\gamma_{0}}{\varphi }\cdot \left[1-{\left(1-P_{e}\left(N_{d}=0\right)\right)}^{M}\right]+\frac{\gamma_{0}}{\varphi }\cdot \left[1-{\left(1-P_{e}\left(N_{d}=1\right)\right)}^{M}\right], \end{array}$$

*where $P_{e}\left(N_{d}=k\right)={\left(\frac{2}{\pi R^{2}}\right)}^{M}\int_{D}^{D+2R}\exp\left(-\beta_{p}{l_{0}}^{\alpha }\sigma^{2}\right)l_{0}{Z(l_{0})}^{M-1}W^{\prime }\left(l_{0},k\right)\arccos\left[\frac{D}{l_{0}}+\frac{{l_{0}}^{2}-D^{2}}{2l_{0}\left(R+D\right)}\right]dl_{0}$,*

*$\beta_{p}=\frac{\beta }{P_{t}}$, $V_{1}\left(l_{0}\right)=\beta_{p}{l_{0}}^{\alpha }P_{j}G_{s}$, $V_{2}\left(l_{0}\right)=\beta_{p}{l_{0}}^{\alpha }P_{j}G_{n}$, $Z\left(l_{0}\right)=\int_{D}^{D+2R}(\frac{l^{\alpha +1}}{l^{\alpha }+\beta {l_{0}}^{\alpha }})\arccos\left[\frac{D}{l}+\frac{l^{2}-D^{2}}{2l(R+D)}\right]dl$,* *$\gamma_{0}=\frac{\theta_{m}}{2}-\arcsin\left(R\sin\left(\frac{\theta_{m}}{2}\right)/L\right)$,* 
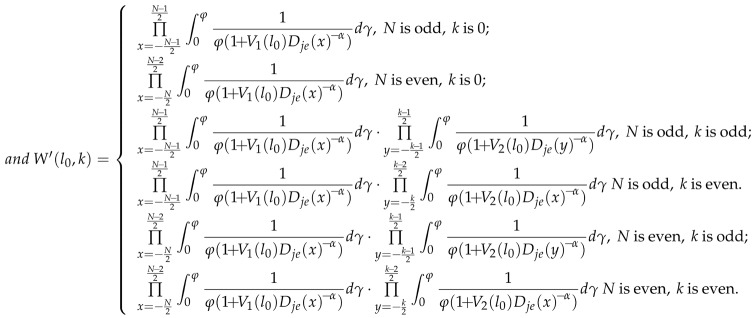

**Proof.**  We present the proof of Theorem 6 in Appendix C. ☐

## 5. Results

In this section, we present the simulation results of probability of successful transmission $P_{T}$ and probability of eavesdropping attack $P_{E}$ considering the NFJ, OFJ and DFJ schemes. The simulation results are generated via Monte Carlo simulations with 50,000 runs and the parameters are given in Table 2.

**Figure 5:**
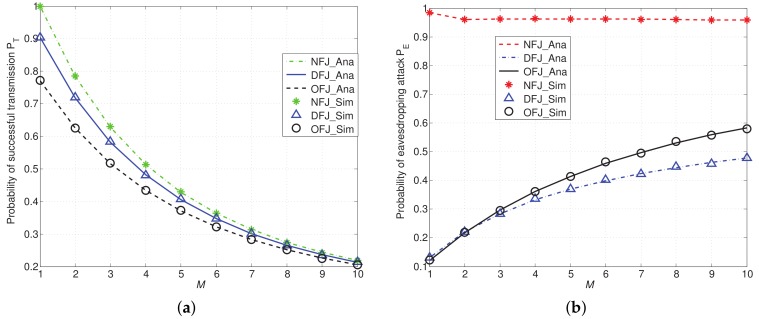
$P_{T}$ and $P_{E}$ with DFJ scheme and OFJ scheme versus NFJ scheme when α=4, D=10, N=9 and *M* varies from 1 to 10. (**a**) Probability of successful transmission $P_{T}$; (**b**) Probability of eavesdropping attack $P_{E}$.

In Figure 5, we present the numerical and simulation results of probability of successful transmission $P_{T}$ and probability of eavesdropping attack $P_{E}$ with different schemes. From Figure 5a, we find $P_{T}$ decreases when the number of legitimate transmitters denoted by *M* increases. Since the receiver only receives the information from the protected transmitter, the cumulative interference from legitimate transmitters to the receiver increases with *M*. When we introduce friendly jammers into the network, compared with the NFJ scheme, $P_{T}$ decreases. The performance of $P_{T}$ with DFJ scheme is better than $P_{T}$ with OFJ scheme. This is because the lower antenna gain of side lobe in the DFJ scheme leads to less interference to the legitimate transmission.

In Figure 5b, the red curve represents the probability of eavesdropping attack $P_{E}$ of the NFJ scheme. From numerical results, we find that the red line decreases very slowly, especially when *M* is larger than 2. For example, when M=4, $P_{E}$ of NFJ scheme is 0.9626, while $P_{E}$ becomes 0.9619 and 0.9602 when *M* becomes 6 and 8, respectively. This result lies in the fact that the eavesdropper may eavesdrop any one of the *M* legitimate transmitters, rather than a specially appointed transmitter. When *M* increases, the interference on the eavesdropper increases. However, the eavesdropper may tap more transmitters as the total number of transmitters is increased. In addition, the performance of $P_{E}$ of DFJ scheme is still better than that of the OFJ scheme, because of the higher antenna gain of main lobe.

From Figure 5a,b we find that introducing friendly jammers into the network will lead to the decrement on both $P_{T}$ and $P_{E}$. However, the influence of friendly jammers on $P_{E}$ is more obvious than the influence on $P_{E}$. For example, when M=5, compared with the NFJ scheme, the reduction of $P_{T}$ with OFJ scheme is 0.0574 (i.e., 13.4% reduced), while the reduction of $P_{E}$ is 0.5485 (i.e., 56.99% reduced). When M=5, compared with the NFJ scheme, the reduction of $P_{T}$ with DFJ scheme is 0.023 (i.e., 5.4% reduced), the reduction of $P_{E}$ is 0.5935 (i.e., 61.66% reduced). Therefore, the DFJ scheme can reduce the probability of eavesdropping attacks more significantly while maintaining the lower impairment to the legitimate communications than the OFJ scheme. This result implies that using friendly jammers can reduce the eavesdropping attack without causing obvious damage on legitimate transmission.

**Figure 6:**
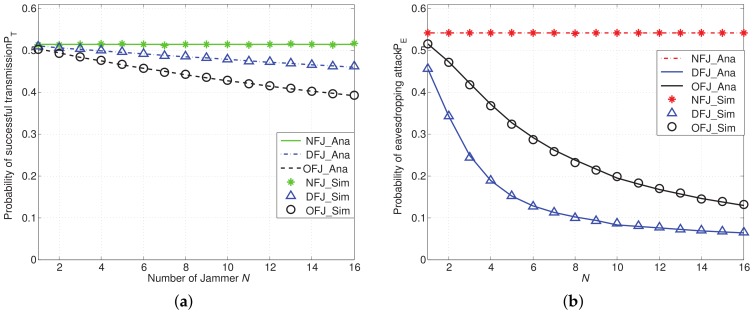
$P_{T}$ and $P_{E}$ with the DFJ scheme and the OFJ scheme versus the NFJ scheme when α=4, D=10, M=4 and *N* varies from 1 to 16. (**a**) Probability of successful transmission $P_{T}$; (**b**) Probability of eavesdropping attack $P_{E}$.

Figure 6 shows the comparison of $P_{T}$ and $P_{E}$ in different schemes with the varied number of friendly jammers denoted by *N*. In Figure 6a, we find that both $P_{T}$ of the DFJ scheme and that of the OFJ scheme decrease when *N* increases. The decrement of $P_{T}$ lies in the increased cumulative interference from friendly jammers. Moreover, Figure 6b shows that $P_{E}$ decreases rapidly when the number of friendly jammers *N* increases, especially when friendly jammers are equipped with directional antennas (i.e., in DFJ scheme). This result can help to verify the effectiveness of OFJ and DFJ schemes in reducing the probability of eavesdropping attacks $P_{E}$.

The results as shown in Figure 6 imply that it may not be necessary to deploy too many friendly jammers in the network. In particular, in the DFJ scheme, we can significantly reduce the probability of eavesdropping attacks while only slightly impairing legitimate communications by introducing a few friendly jammers. For example, when N=8, compared with NFJ scheme, the reduction of $P_{T}$ in DFJ scheme is 0.0291 (i.e., 5.66% reduction), while the reduction of $P_{E}$ in the DFJ scheme is 0.4399 (i.e., 81.24% reduction).

**Figure 7:**
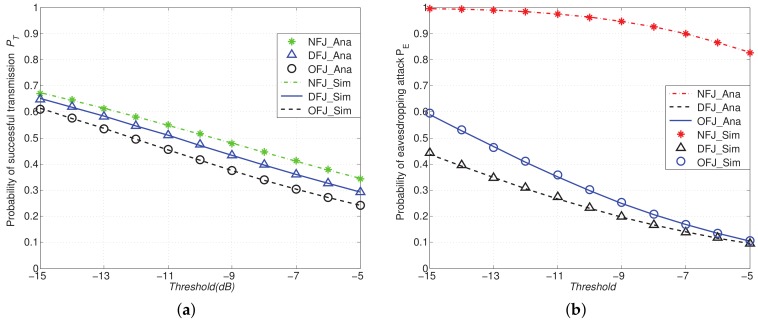
$P_{T}$ and $P_{E}$ with DFJ scheme and OFJ scheme versus NFJ scheme when α=4, D=10, M=4, N=9, SINR threshold *T* and β varies from −15dB to −5dB. (**a**) Probability of successful transmission $P_{T}$; (**b**) Probability of eavesdropping attacks $P_{E}$.

Figure 7 shows the comparison of $P_{T}$ and $P_{E}$ with DFJ, OFJ schemes versus the NFJ scheme with different SINR threshold. It is shown in Figure 7 that $P_{T}$ decreases with the threshold *T* and $P_{E}$ decreases with the threshold β. From Figure 7a, similarly to Figure 5b, we find that using friendly jammers can always reduce the probability of eavesdropping attacks $P_{E}$ compared with the NFJ scheme, and the DFJ scheme performs better than the OFJ scheme obviously.

**Figure 8:**
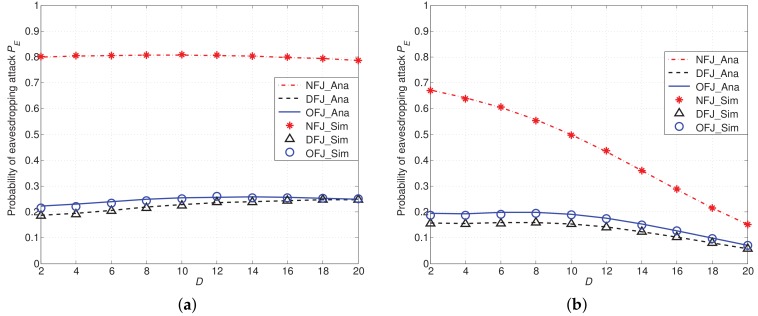
Probability of eavesdropping attacks $P_{E}$ with DFJ scheme and OFJ scheme versus NFJ scheme when α=3,4 with distance D ranging from 2 to 20. (**a**) α=3; (**b**) α=4.

In another set of simulations as presented in Figure 8, we compare the probability of eavesdropping attack $P_{E}$ of different schemes with varied distance between the eavesdropper and the network boundary *D*. From Figure 8a, we can find that $P_{E}$ of NFJ, OFJ and DFJ schemes vary slightly when path loss factor α=3. It means when α=3, path loss effect has no obvious impact on $P_{E}$. This result lies in the fact that path loss has the influence on both useful signal and interference. However, when the path loss factor α increases from 3 to 4, as shown in Figure 8b, $P_{E}$ of three schemes decreases rapidly, especially in the NFJ scheme. This result implies that the path loss has a more obvious influence on useful signal than that on interference. From Figure 8, we also find that using friendly jammers can reduce the probability of eavesdropping attacks $P_{E}$ compared with the NFJ scheme.

## 6. Discussions

In this section, we first discuss the impact of our friendly jamming schemes when the eavesdropper is located inside the network. Then we discuss the impact of our friendly jamming schemes on legitimate transmissions in other networks.

### 6.1. Impact on the Eavesdropper Inside of Network

**Figure 9:**
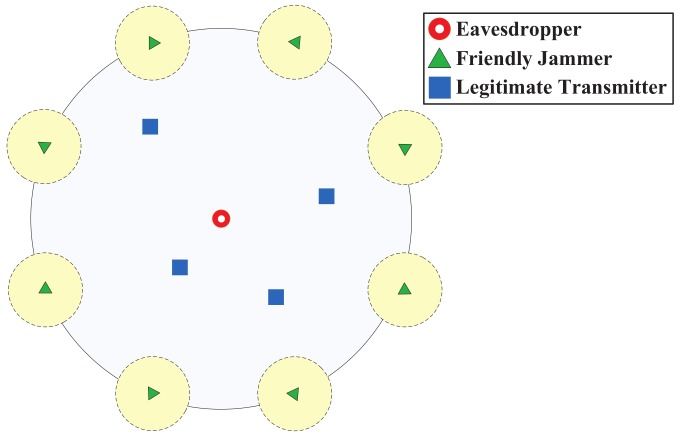
Eavesdropper inside of the network.

In Section 4, we analyze the impact of friendly jamming schemes on the eavesdropper who is prevented from entering the protected area. It is feasible in a practical industrial environment that the eavesdropper has the difficulty of entering the protected network area (e.g., the barbed wire entanglement around a plant). However, we can also apply our previous results in [27] to analyze the scenario in which an eavesdropper enters the network area. In particular, we consider that friendly jammers are regularly placed at deterministic locations [27] and the eavesdropper is located at the center of this area, as shown in Figure 9. We then apply the general theoretical models presented in [27] to derive the eavesdropping probability.

Regarding the case in which the eavesdropper is not located at the center of the network, we can first calculate the cumulative distance from the eavesdropper to each of friendly jammers via the approach proposed in [26] and used [28]. We can then derive the impact of friendly jammers on the eavesdropper inside of network by following the similar steps in [27] and plugging in the cumulative distance. Due to the space limitation and the similarity to our previous method [27], we ignore the derivation of the eavesdropping probability in the scenario that an eavesdropper enters the network.

### 6.2. Impact on Legitimate Transmissions in Other Networks

In Section 3, we investigated the impact of friendly jamming schemes on legitimate transmissions in our protected network area. Since our friendly jammers are deployed on the boundary of our protected transmission area, the networks near this area may possibly be interfered with by our friendly jamming schemes. Therefore, we next investigate the impacts of friendly jamming schemes on legitimate transmissions in other networks.

**Figure 10:**
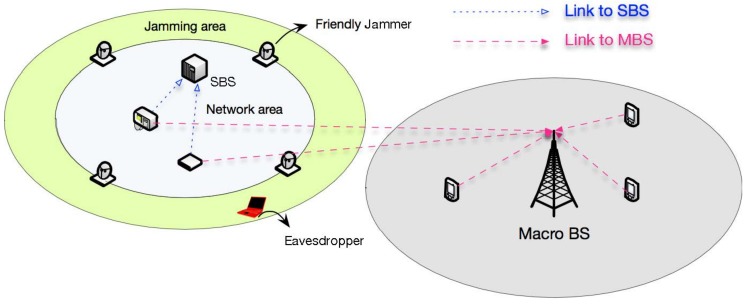
Impact of friendly jammers on other networks.

In Figure 10, we show the relationship between our protected network (at the left hand side) and another network nearby (at the right hand side). It is worth mentioning that a crowdsensing device in our protected network often has the multi-homing capability [29], i.e., accessing two different networks (e.g., a small cell and a macro cell). The impact of our friendly jamming schemes on legitimate transmissions of another network can be analyzed according to two different scenarios: (1) both protected network and another network are using different channels; (2) both protected network and another network are using the same channel. In the first scenario in which different frequencies are allocated to the protected network and another network. In this scenario, the interference of our friendly jamming schemes is negligible on legitimate transmissions in other networks.

Then we analyze the second scenario. In this scenario, both the protected network and another network are using different channels. It is obvious that the analytical result in Section 4 can be trivially used to investigate the interference from friendly jammers on legitimate transmissions in another network. In particular, the interference generated by the OFJ scheme on legitimate transmissions outside the network is given by Lemma 1 in Section 4. The interference from the DFJ scheme on legitimate transmissions outside the network is given by Lemma 2 in Section 4.

Another concern related to our friendly jamming schemes is legitimacy. For example, jamming schemes are restricted in US and Europe. In Europe, the transmitting power of jammers is limited to be less than 20 dBm for 2.4 GHz band [30]. Therefore, we can either *limit the jamming range* or *restrict the jamming* period so that the impact on other legitimate communications will be minimized. The analytical results of OFJ and DFJ imply that the intensity of interference generated by our friendly jamming scheme heavily relies on the channel factors, for example, the transmitting power of jammers, antenna gain, path loss, etc. Therefore, we can adjust the transmitting power and the antenna gain of friendly jammers so that the jamming range can be minimized. Another approach of limiting the impact of friendly jamming schemes is restricting the time of the emitting jamming signal. For example, we can only send the jamming signal at the crucial stages (e.g., key generation phase [31] or vulnerable phase [14]).

## 7. Conclusions

In this paper, we propose a novel friendly jamming scheme to protect confidential communications from eavesdropping attacks. To evaluate the effectiveness of our scheme, we establish a theoretical model to analyze the probability of eavesdropping attacks and the probability of successful transmission. Moreover, we verify our model with extensive simulations. The agreement between analysis and simulation results verifies the accuracy of our analysis.

Our results show that our scheme can significantly decrease the eavesdropping risk compared with the no friendly jamming scenario and meanwhile that it maintains low decrease on the transmission probability. In addition, we find that using directional antennas compared with omni-directional antennas on friendly jammers can further decrease the eavesdropping risk while obviously mitigating the influence on the transmission probability.

## Figures and Tables

**Table 1:** Notation Summary.

Notation	Description
*R*	Radius of protected circular legitimate communication area
*D*	Distance between eavesdropper to the boundary of protected circular area
$P_{t},P_{j}$	Transmission power of legitimate user and friendly jammer
l,r	Distance between the legitimate transmitter and eavesdropper/legitimate receiver
*h*	Fading random variable
α	Path loss exponent
Φ,λ	Point process and intensity of legitimate users
T,β	SINR threshold for a successful legitimate transmission/eavesdropping attack
*M*	Expectation of the number of legitimate transmitters
*N*	Number of friendly jammers
E(X)	Expectation of random variable *X*
$G_{m},G_{s}$	Antenna gain of main lobe, antenna gain of side lobe
$\theta_{m}$	Main lobe beamwidth of the directional antenna
$G_{t},G_{e},G_{j}$	Antenna gain of the legitimate transmitters/eavesdropper/friendly jammers
$P_{E}$	Probability of eavesdropping attacks
$P_{e}$	Probability of eavesdropping a certain transmitter successfully
$P_{T}$	Probability of successful transmission
$I_{t},I_{j}$	Cumulative interference from legitimate transmitters/friendly jammers on the receiver
$I_{t}e,I_{j}e$	Cumulative interference from legitimate transmitters/friendly jammers on the eavesdropper
$\sigma^{2}$	Noise power of Gaussian Addictive White Noise

**Table 2:** Notation and parameters.

Parameters	Values
Radius of protected communication area *R*	20
Transmission power of legitimate users $P_{t}$	20 dBm
Transmission power of friendly jammers $P_{j}$	20 dBm
Noise power	−90 dBm
Antenna gain of main lobe $G_{m}$	10 dBi
Main lobe beamwidth $\theta_{m}$	$\frac{\pi }{3}$

## Appendix A

**Proof** **of** **Lemma** **1.** From the relationship of $\Delta EJ_{1}O$ in Figure 3, we can calculate the distance between jammer $J_{1}$ and the eavesdropper *E* in the following equation,

$$D_{r}\left(1\right)={\left[R^{2}+L^{2}-2RL\cos\left(2\varphi -\gamma \right)\right]}^{\frac{1}{2}}.$$

From the relationship of $\Delta EJ_{2}O$, we get the distance between eavesdropper E and jammer $J_{2}$:

$$D_{l}\left(1\right)={\left[R^{2}+L^{2}-2RL\cos\gamma \right]}^{\frac{1}{2}}.$$

Following the similar approach, we have the distance between eavesdropper E and the *m*th nearest jammer at the right-hand-side of jammer $J_{1}$ as follows,

(A1) $$D_{r}\left(m\right)={\left[R^{2}+L^{2}-2RL\cos\left(2m\varphi -\gamma \right)\right]}^{\frac{1}{2}},$$

and the distance between eavesdropper E and the *n*th nearest jammer at the left-hand-side of jammer $J_{1}$ as follows,

(A2) $$D_{l}\left(n\right)={\left[R^{2}+L^{2}-2RL\cos\left[2\left(n-1\right)\varphi +\gamma \right]\right]}^{\frac{1}{2}}.$$

Combining Equations (A1) and (A2), we can have the expression of the distance between jammers and eavesdropper $D_{je}\left(x\right)$ as follows,

(A3) $$D_{je}\left(x\right)={\left[R^{2}+L^{2}-2RL\cos\left(2x\varphi +\gamma \right)\right]}^{\frac{1}{2}},$$

where *x* is an integer whose range depends on *N*. When *N* is odd, $x\in \left[-\frac{N-1}{2},\frac{N-1}{2}\right]$; when *N* is even, $x\in \left[-\frac{N}{2},\frac{N-2}{2}\right]$. Substituting the distance into the channel model, we get the cumulative interference of jammers to the eavesdropper as given in Lemma 1. ☐

## Appendix B

**Proof** **of** **Lemma** **2.** From Section 4.2 we have the distance between eavesdropper and jammers as given in Equation (A3). Then we show the interference of friendly jammers on eavesdroppers according to different cases of $N_{d}$. Case 0: when $N_{d}=0$, the interference of friendly jammers on eavesdroppers is from side lobe of friendly jammers, If *N* is even,

$$I_{je}=I_{js}=P_{j}G_{s}\left[\sum_{x=-\frac{N}{2}}^{\frac{N-2}{2}}h_{x}D_{je}{\left(x\right)}^{-\alpha }\right];$$

If *N* is odd,

$$I_{je}=I_{js}=P_{j}G_{s}\left[\sum_{x=-\frac{N-1}{2}}^{\frac{N-1}{2}}h_{x}D_{je}{\left(x\right)}^{-\alpha }\right].$$

Case 1: when $N_{d}=1$, the interference of friendly jammers on eavesdroppers is

$$I_{je}=P_{j}G_{n}hD_{je}{\left(0\right)}^{-\alpha }+I_{js}.$$

Case 2: when $N_{d}=2$, the interference of friendly jammers on eavesdroppers is

$$I_{je}=P_{j}G_{n}h\left[D_{je}{\left(-1\right)}^{-\alpha }+D_{je}{\left(0\right)}^{-\alpha }\right]+I_{js}.$$

Case 3: when $N_{d}=3$, the interference of friendly jammers on eavesdroppers is

$$I_{je}=P_{j}G_{n}h\left[{D_{je}\left(-1\right)}^{-\alpha }+D_{je}{\left(1\right)}^{-\alpha }+D_{je}{\left(1\right)}^{-\alpha }\right]+I_{js}.$$

Case *k*: when $N_{d}=k$, the interference of friendly jammers on eavesdroppers is If *k* is even,

$$I_{je}=P_{j}G_{n}\left[\sum_{x=-\frac{k}{2}}^{\frac{k-2}{2}}h_{x}D_{je}{\left(x\right)}^{-\alpha }\right]\;+\;I_{js},$$

If *k* is odd,

$$I_{je}=P_{j}G_{n}\left[\sum_{x=-\frac{k-1}{2}}^{\frac{k-1}{2}}h_{x}D_{je}{\left(x\right)}^{-\alpha }\right]\;+\;I_{js}.$$

Therefore, we obtain the expression of $I_{\mathrm{je}}$ in Lemma 2 by integrating the above cases. ☐

## Appendix C

**Proof** **of** **Theorem** **6.** According to the definition of the eavesdropping probability $P_{E}$, we need to derive the probability $P_{e}$ first. The derivation of eavesdropping probability $P_{e}$ in DFJ scheme is similar to the derivation OFJ scheme in Theorem 5, while the main difference is the cumulative interference from friendly jammers. Therefore, we have the following expressions:

(A4) $$\begin{array}{c} P_{e}\left(N_{d}=k\right)=\int_{D}^{D+2R}\frac{2l_{0}}{\pi R^{2}}P[h\geq \beta_{p}{l_{0}}^{\alpha }\left(\sigma^{2}+I_{te}+I_{je}\left(k\right)\right)|l_{0}]\cdot \arccos\left(\frac{d}{l_{0}}+\frac{{l_{0}}^{2}-d^{2}}{2l_{0}\left(R+d\right)}\right)dl_{0}, \end{array}$$

where $\beta_{p}=\frac{\beta }{P_{t}}$, and

(A5) $$\begin{array}{cc} P & \left[h_{0}\geq \beta_{p}{l_{0}}^{\alpha }\left(\sigma^{2}+I_{te}+I_{je}\left(k\right)\right)|l_{0}\right]=e^{-\beta_{p}{l_{0}}^{\alpha }\sigma^{2}}\cdot E_{I_{te}}\left[\exp\left(-\beta_{p}{l_{0}}^{\alpha }I_{te}\right)\right]\cdot E_{I_{je}\left(k\right)}\left[\exp\left(-\beta_{p}{l_{0}}^{\alpha }I_{je}\left(k\right)\right)\right]. \end{array}$$

The influence of legitimate transmitters on the eavesdropper remains unchanged. Therefore, we have the $E_{I_{te}}\left[\exp\left(-\beta_{p}{l_{0}}^{\alpha }I_{te}\right)\right]$ given in Equation (24). From the expression of interference from directional jammers on eavesdropper as given in the Lemma 2, we have  where $V_{1}\left(l_{0}\right)=\beta_{p}{l_{0}}^{\alpha }P_{j}G_{s}$, $V_{2}\left(l_{0}\right)=\beta_{p}{l_{0}}^{\alpha }P_{j}G_{n}$ and $G_{n}=G_{m}-G_{s}$. After plugging Equations (25) and (A6) into the Equation (A5), and substituting the new expression of Equation (A5) into Equation (A4), we obtain the result of $P_{e}$ in the following expression,

(A7) $$P_{e}\left(N_{d}=k\right)={\left(\frac{2}{\pi R^{2}}\right)}^{M}\cdot \int_{D}^{D+2R}\exp\left(-\beta_{p}{l_{0}}^{\alpha }\sigma^{2}\right)l_{0}{Z(l_{0})}^{M-1}W^{\prime }\left(l_{0},k\right)\arccos\left[\frac{D}{l_{0}}+\frac{{l_{0}}^{2}-D^{2}}{2l_{0}\left(R+D\right)}\right]dl_{0}.$$

Then we derive $P_{E}$ in the DJF scheme. When there are *k* jammers interfering with the eavesdropper via main lobes, according to Total Probability Theorem, we have the following expression,

(A8) $$P_{E}=\sum_{x=0}^{k}P\left(N_{d}=x\right)\cdot P_{E}\left(N_{d}=x\right).$$

However, the premise of k>1 is that both the number of jammers and the distance *D* between eavesdropper and the communication area are large. Since this situation seldom exists in real communication environment, we simplify the expression of $P_{E}$ by considering only two scenarios: k=0 and k=1. When there is more than one jammer interfering with the eavesdropper via its main lobe, we assume there is one jammer interfering with the eavesdropper via its main lobe. Then we have the upper bound of $P_{E}$ as follows,

(A9) $$\begin{array}{c} P_{E}\approx P\left(N_{d}=0\right)\cdot P_{E}\left(N_{d}=0\right)+P\left(N_{d}=1\right)\cdot P_{E}\left(N_{d}=1\right). \end{array}$$

Since our result of $P_{E}$ is a upper bound, the effect of the DFJ scheme on the eavesdropper in reality will be more highlighted. From the geometrical relationship as given in Figure 3, we have $\gamma =\omega -\arcsin\left(R\sin\omega /L\right)$. We denote $\gamma_{0}=\frac{\theta_{m}}{2}-\arcsin\left(R\sin\left(\frac{\theta_{m}}{2}\right)/L\right)$. When $\gamma >\gamma_{0}$, we have $\omega >\frac{\theta_{m}}{2}$, which means the eavesdropper locates in area $A_{0}$ and no jammer interferes with the eavesdropper via its main lobe. Since the degree γ is uniformly distributed, the result in Equation (A9) becomes

(A10) $$\begin{array}{c} P_{E}=\frac{\varphi -\gamma_{0}}{\varphi }\cdot \left[1-{\left(1-P_{e}\left(N_{d}=0\right)\right)}^{M}\right]+\frac{\gamma_{0}}{\varphi }\cdot \left[1-{\left(1-P_{e}\left(N_{d}=1\right)\right)}^{M}\right], \end{array}$$

where $P_{e}\left(N_{d}=0\right)$ and $P_{e}\left(N_{d}=1\right)$ can be calculated from Equation (A7). ☐
